# Systematic investigation of the underlying mechanisms of GLP-1 receptor agonists to prevent myocardial infarction in patients with type 2 diabetes mellitus using network pharmacology

**DOI:** 10.3389/fphar.2023.1125753

**Published:** 2023-02-14

**Authors:** Guorong Deng, Jiajia Ren, Ruohan Li, Minjie Li, Xuting Jin, Jiamei Li, Jueheng Liu, Ya Gao, Jingjing Zhang, Xiaochuang Wang, Gang Wang

**Affiliations:** ^1^ Department of Critical Care Medicine, the Second Affiliated Hospital of Xi’an Jiaotong University, Xi’an, China; ^2^ Department of Cardiology, The Second Affiliated Hospital of Shaanxi University of Traditional Chinese Medicine, Xi’an, China

**Keywords:** GLP-1 receptor agonists, type 2 diabetes mellitus, myocardial infarction, network pharmacology, atheromatous plaque, myocardial remodeling, thrombosis

## Abstract

**Background:** Several clinical trials have demonstrated that glucagon-like peptide-1 (GLP-1) receptor agonists (GLP-1RAs) reduce the incidence of non-fatal myocardial infarction (MI) in patients with type 2 diabetes mellitus (T2DM). However, the underlying mechanism remains unclear. In this study, we applied a network pharmacology method to investigate the mechanisms by which GLP-1RAs reduce MI occurrence in patients with T2DM.

**Methods:** Targets of three GLP-1RAs (liraglutide, semaglutide, and albiglutide), T2DM, and MI were retrieved from online databases. The intersection process and associated targets retrieval were employed to obtain the related targets of GLP-1RAs against T2DM and MI. Gene Ontology (GO) and Kyoto Encyclopedia of Genes and Genes (KEGG) enrichment analyses were performed. The STRING database was used to obtain the protein-protein interaction (PPI) network, and Cytoscape was used to identify core targets, transcription factors, and modules.

**Results:** A total of 198 targets were retrieved for the three drugs and 511 targets for T2DM with MI. Finally, 51 related targets, including 31 intersection targets and 20 associated targets, were predicted to interfere with the progression of T2DM and MI on using GLP-1RAs. The STRING database was used to establish a PPI network comprising 46 nodes and 175 edges. The PPI network was analyzed using Cytoscape, and seven core targets were screened: AGT, TGFB1, STAT3, TIMP1, MMP9, MMP1, and MMP2. The transcription factor MAFB regulates all seven core targets. The cluster analysis generated three modules. The GO analysis for 51 targets indicated that the terms were mainly enriched in the extracellular matrix, angiotensin, platelets, and endopeptidase. The results of KEGG analysis revealed that the 51 targets primarily participated in the renin-angiotensin system, complement and coagulation cascades, hypertrophic cardiomyopathy, and AGE-RAGE signaling pathway in diabetic complications.

**Conclusion:** GLP-1RAs exert multi-dimensional effects on reducing the occurrence of MI in T2DM patients by interfering with targets, biological processes, and cellular signaling pathways related to atheromatous plaque, myocardial remodeling, and thrombosis.

## Introduction

Over the past four decades, the number of people living with diabetes has increased from 108 million in 1980 to 537 million in 2021, of which the overwhelming majority (over 90%) were diagnosed as type 2 diabetes mellitus (T2DM). In 2021, 6.7 million deaths were caused by diabetes or its complications (International Diabetes Federation, 2022). Among the extensive T2DM-related complications, acute myocardial infarction is a life-threatening and severe complication (Rosenblit, 2019). More than one-third of T2DM patients with myocardial infarction (MI) die within 10 years, and long-term all-cause mortality and cardiovascular mortality are even higher in younger patients than in elderly patients (Singh et al., 2020; Zheng et al., 2021). Numerous studies have shown that strict glycemic control promotes a decrease in non-fatal MI (Rodriguez-Gutierrez et al., 2019). However, intensive controls are followed by severe side effects, such as hypoglycemia; therefore, effective and safe methods for controlling glycemic levels, while simultaneously reducing risk factors for MI, act as necessary interventions in treating patients with T2DM.

Glucagon-like peptide-1 receptor agonists (GLP-1RAs), such as liraglutide and dulaglutide, are widely used to treat patients with T2DM and obesity. They exert beneficial effects, including inhibition of glucagon secretion, delayed gastric emptying, decreased appetite, rare occurrence of hypoglycemia, and controlled weight gain (Helmstadter et al., 2022). In recent years, four clinical trials have shown that dulaglutide (REWIND trial) (Gerstein et al., 2019), albiglutide (HARMONY trial) (Hernandez et al., 2018), semaglutide (SUSTAIN-6 trial) (Marso et al., 2016a), and liraglutide (LEADER trial) (Marso et al., 2016b) have cardiovascular benefits in patients with T2DM, including reducing the occurrence of non-fatal MI. A meta-analysis reported that patients with T2DM benefited from different GLP-1RAs in terms of major adverse cardiac events, all-cause mortality, hospital admission for heart failure, and renal function (Sattar et al., 2021). However, although GLP-1RA therapies are approved and considered safe for treating patients with T2DM, the exerted cardiovascular protection mechanism is still not fully clear. An increasing number of studies have demonstrated that the GLP-1 receptor is expressed in numerous types of cells, including those in the cardiovascular tissues, such as endothelial cells of the left ventricle (GTExPortal, 2021). Theoretically, GLP-1 binds to its receptor, stimulating the adenylyl cyclase pathway, and leading to insulin synthesis and release. As the treatment for T2DM may not fully explain the cardiovascular protective effects of GLP-1RAs, these still must be comprehensively investigated.

**FIGURE 1:**
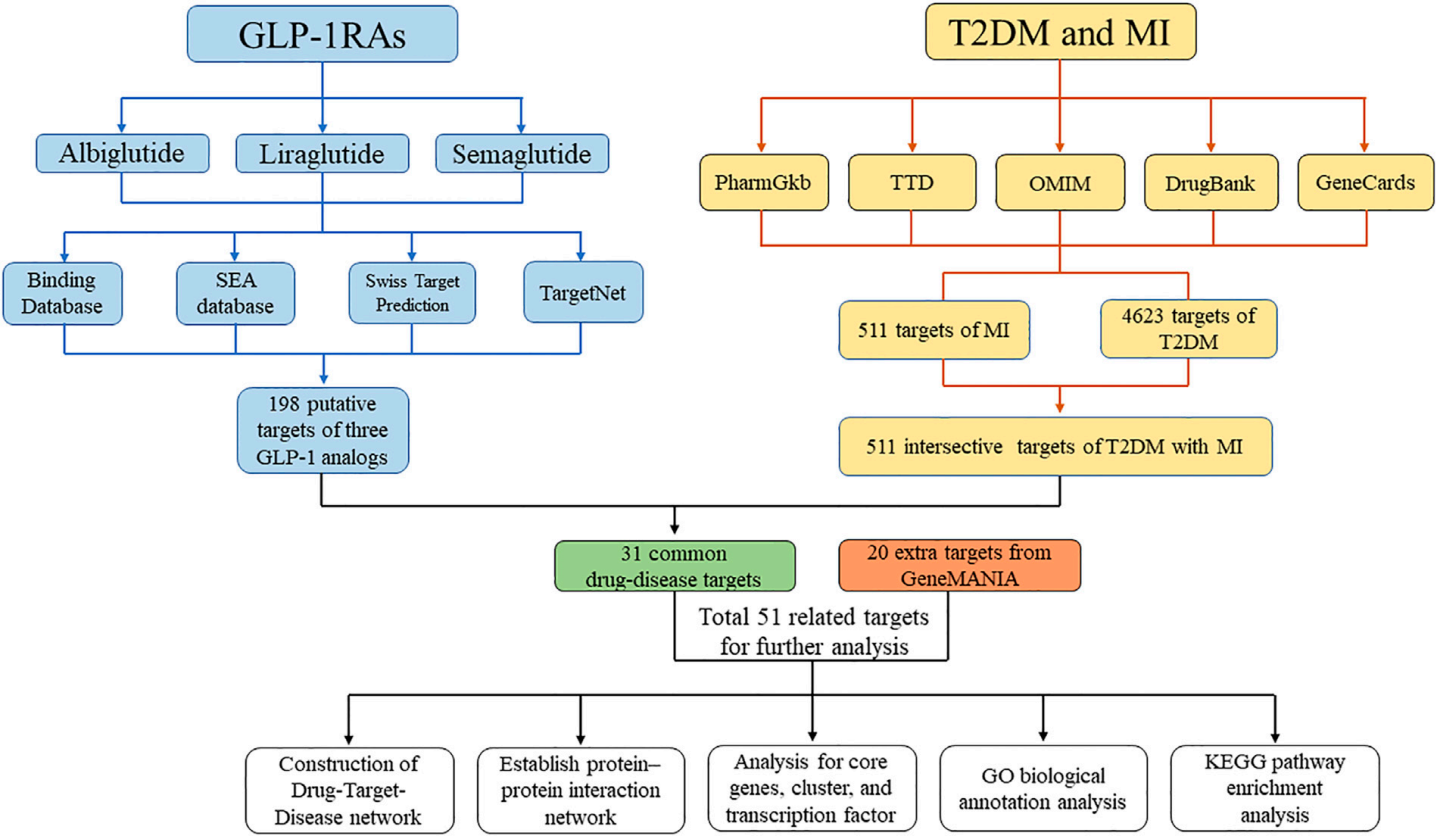
Flow chart for the process of the study. The flow chart shows the process of investigating the pharmacology mechanism of GLP-1RAs against T2DM and MI.

Network pharmacology is a big data integration method based on numerous databases and statistical algorithms (Hong et al., 2021). It aims to investigate diseases at the systemic level and define the interaction between drugs and the body based on the equilibrium theory of biological networks (Zhang, 2016). Chronic diseases are generally caused by a complicated dysfunction of a related regulatory network instead of a single protein or gene (Nogales et al., 2022). Based on an integrated research strategy, the network pharmacology method provides a more efficient and convenient system for determining the relationship between drugs and diseases. In this study, we applied an integrated research strategy to investigate the mechanism of specific GLP-1RAs in T2DM and MI, which may provide a comprehensive interpretation of the cardiovascular protective effect of GLP-1RAs. A flow chart of the study process is shown in Figure 1.

## Materials and methods

### Target prediction for GLP-1 agonists

The chemical structures (mainly in SMILES format) of three GLP-1Ras (liraglutide, semaglutide, and albiglutide) were retrieved from PubChem, an open chemistry database at the National Institutes of Health (https://pubchem.ncbi.nlm.nih.gov). As dulaglutide does not have a defined chemical structure, it was excluded from our study. Next, the following four target prediction databases were selected to retrieve targets for the GLP-1RAs: (1) The Binding Database (http://www.bindingdb.org/bind/ByTargetNames.jsp), a public and web-accessible database containing 2,096,653 binding data points for 8,185 proteins and over 920,703 drug-like molecules (Gilson et al., 2016); (2) The SEA database (https://sea.bkslab.org/), which can be rapidly used to search large compounds and to build cross-target similarity maps (Keiser et al., 2007); (3) The Swiss Target Prediction (http://www.swisstargetprediction.ch/) that allows estimating the most probable protein targets of a small molecule (Daina et al., 2019), and (4) the TargetNet (http://targetnet.scbdd.com/home/index/), an open web server that can be used to predict the binding of multiple targets for any given molecule across 623 proteins by establishing a high-quality model for each human protein (Yao et al., 2016). All targets from the four databases were further standardized into official gene symbols using Universal Protein Resource (https://www.uniprot.org/) (Consortium, 2021) for subsequent analysis.

## Target collection for T2DM and MI

With the keywords “type 2 diabetes,” “type 2 diabetes mellitus,” “myocardial infarction,” “non-fatal myocardial infarction,” “acute myocardial infarction,” “ST-segment elevation myocardial infarction,” and “non-ST-segment elevation myocardial infarction,” the target genes associated with T2DM and MI were retrieved from the PharmGkb (https://www.pharmgkb.org/), TTD (http://db.idrblab.net/ttd/), GeneCards (https://www.genecards.org), DrugBank (https://go.drugbank.com/), and OMIM (https://www.omim.org) databases. The PharmGKB database is a pharmacogenomic knowledge resource containing clinical information (Whirl-Carrillo et al., 2021). The TTD database provides information about the known and explored therapeutic protein and nucleic acid targets, the targeted disease, pathway information, and the corresponding drugs directed at each target (Wang et al., 2020). The GeneCards database integrates gene-centric data from more than 150 web sources, including genomic, transcriptomic, proteomic, genetic, clinical, and functional information (Stelzer et al., 2016). The DrugBank database contains information regarding drugs and drug targets (Wishart et al., 2018). The OMIM database is a comprehensive and authoritative compendium of human genes and genetic phenotypes. The five databases provided comprehensive and complementary resources for obtaining targets for the diseases. All T2DM and MI targets from the five databases were transformed into the official gene symbol format.

### Related targets

The targets of GLP-1RAs, T2DM, and MI were uploaded to an online Venn diagram tool (http://www.bioinformatics.com.cn/static/others/jvenn/example.html) to obtain a Venn diagram showing the intersection targets of GLP-1RAs against T2DM and MI. Then, the GeneMANIA database was applied to find extra targets, highly associated with the intersection targets, using a massive set of functional association data (Warde-Farley et al., 2010). Finally, these targets and intersecting targets were integrated into a set of related targets for further analysis.

### Construction of the drug-target-disease network

The relationship among the two diseases, three GLP-1RAs, and extra targets was established using Microsoft Excel and then input into Cytoscape (Version 3.8.2) to build and visualize a drug-target-disease network presented by nodes and edges. Nodes represent drugs, diseases, and target genes, whereas edges represent the existing correlations between any two nodes.

### Protein-protein interaction (PPI) network data

The related targets were uploaded to the STRING database (https://string-db.org/) and processed in a multiple protein analysis pattern to obtain the PPI network data. The STRING database focuses on researching the interactive relationships between proteins, which helps identify core regulatory genes (Szklarczyk et al., 2019). Some key parameters were also set. For example, the organism was chosen as *Homo sapiens*; the interaction score was selected as high confidence of 0.7, and disconnected nodes were hidden in the network. Finally, the network data were downloaded in a “TSV” format file for further analysis, and a visualized network image was obtained.

### Gene Ontology (GO) and Kyoto Encyclopedia of genes and genomes (KEGG) enrichment analysis

To further uncover the underlying biological process and involved signaling pathways in related targets, the KEGG pathway and GO enrichment analysis, including biological process (BP), cellular component (CC), and molecular function (MF), were conducted using Enrichr web tools (Kuleshov et al., 2016), and these enrichment results were presented in a scatter plot using the Appyters web application (Clarke et al., 2021). Similar gene sets were clustered in a scatter plot using the Leiden algorithm. According to the q value (adjusted *p*-value), the top five GO and KEGG analysis terms were listed and marked in the scatter plots.

### PPI network analysis

The “TSV” file of the PPI network data was input to the Cytoscape software to identify hub targets and clusters using the Cytohubba (Version 0.1) and MCODE (Version 2.0.0) plugins, respectively. The Cytohubba plugin provides 11 methods for exploring virtual nodes in biological networks (Chin et al., 2014). Referring to the method from *Shen Jiayu et al.*, the maximal clique centrality (MCC), edge percolated component (EPC), maximum neighborhood component (MNC), and degree algorithms (Shen et al., 2019) were selected in this study to generate four values for each target, calculate a mean value for each algorithm, and finally select the targets for which the values were simultaneously higher than the mean values of each algorithm as the core targets. The Iregulon (Version 1.3) plugin was used to identify the direct transcription factor of core targets (Janky et al., 2014). The MCODE plugin can mine protein complexes or functional modules from complex protein networks (Bader and Hogue, 2003). All the processing parameters were set to the default values. Additionally, the most important node in the cluster, SEED node, may be the critical target of each cluster. Next, the targets in each cluster were subjected to KEGG pathway enrichment analysis using the Enrichr web tool.

## Results

### Potential targets of the agonists/diseases and their related targets

We collected the molecular structures of three GLP-1RAs (liraglutide, semaglutide, and albiglutide) from the PubChem database. The detailed information is listed in Table 1. In total, 210 targets were obtained from the Binding Database: 77 were for albiglutide, 77 for liraglutide, and 56 for semaglutide. A total of 370 targets were identified using the SEA database: 155 for albiglutide, 133 for liraglutide, and 82 for semaglutide. The Swiss database was used to obtain 158 potential targets: 62 for albiglutide, 60 for liraglutide, and 36 for semaglutide. From the TargetNet database, 167 targets were obtained: 61 for albiglutide, 56 for liraglutide, and 50 for semaglutide. Finally, 198 targets remained after the integration and elimination of duplicates.

**TABLE 1:** Chemical information for three GLP-1RAs from the PubChem database.

Order	Name	PubChem ID	Molecular formula	Molecular weight	2D structure
1	Albiglutide	145994868	C_148_H_224_N_40_O_45_	3283.6	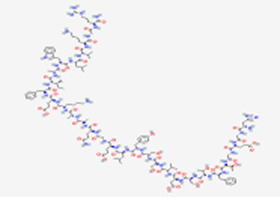
2	Liraglutide	16134956	C_172_H_265_N_43_O_51_	3751	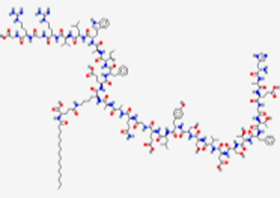
3	Semaglutide	56843331	C_187_H_291_N_45_O_59_	4114	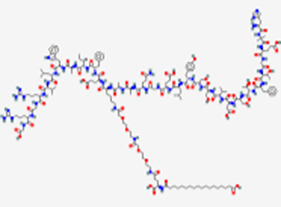

**FIGURE 2:**
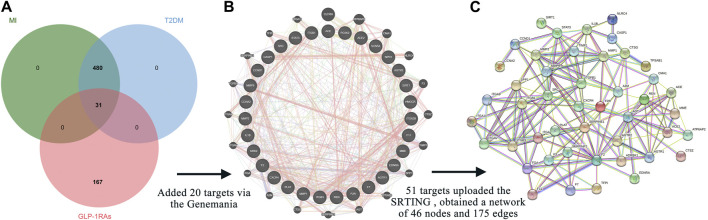
Intersection targets and associated targets constructed the protein-protein interaction (PPI) network. **(A)** The 31 intersection targets overlap between the targets of GLP-1RAs and the targets of T2DM with MI. **(B)** Twenty targets associated with 31 intersection targets were generated *via* the GeneMANIA database. **(C)** The 51 related targets constructed a PPI network containing 46 nodes and 175 edges. The interaction score was set at 0.7 (high confidence) and hid disconnected nodes in the network.

When collecting disease targets, T2DM-related keywords were input into the five databases, and a total of 5004 targets were obtained: 4486 from the GeneCards database, 282 from the OMIM database, 24 from the PharmGkb database, 109 from the TTD database, and 103 from the DrugBank database. Finally, 4623 targets remained after removing repetitions. Parallelly, we also retrieved the MI-related targets from the five databases, and 727 targets were identified, including 313 from the GeneCards database, 18 from the OMIM database, 121 from the PharmGkb database, 47 from the TTD database, and 133 from the DrugBank database. A total of 511 unique targets were identified after removing duplicates. Interestingly, 511 targets of MI were all covered into targets of T2DM. The online Venn diagram tool generated 31 intersection targets between drugs and diseases (Figure 2A).

The GeneMANIA database provided additional 20 targets (Figure 2B), highly associated with intersection targets. Finally, 51 related targets were identified, indicating potential mechanisms to understand how diabetic patients benefit from the three GLP-1RAs in preventing MI. All 51 related targets were uploaded to the STRING database, and a PPI diagram (Figure 2C) and a TSV format file were obtained. The PPI network included 46 nodes and 175 edges.

### Construction of the drugs-diseases-targets network

**FIGURE 3:**
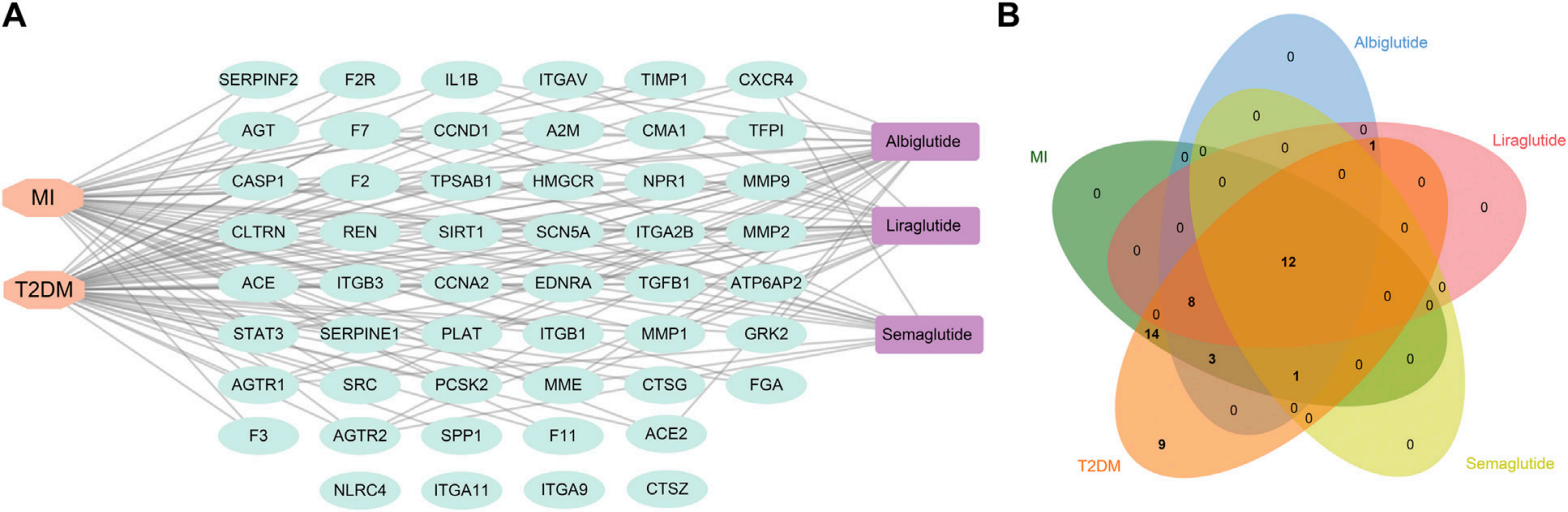
The construction of a drug-target-disease network. **(A)** Network construction of drug-target-disease composed of three drugs (purple), 102 targets (cyan), and two diseases (orange) *via* Cytoscape software. **(B)** Venn diagram shows 12 common targets of three drugs against T2DM and MI, and these targets could be regarded as crucial factors mediating the effects of GLP-1RAs on T2DM and MI.

The three GLP-1RAs, T2DM and MI, and 51 related targets were input into Cytoscape to construct a drug-disease-target network (Figure 3A). The network contains 56 nodes and 114 edges. We found that T2DB was linked to 47 related targets and MI to 38 targets. Furthermore, 25 connections linked albiglutide to all targets, 21 to liraglutide, and 13 to semaglutide. In contrast, four nodes (NLRC4, ITGA11, ITGA9, and CTSZ) were not associated with any agonists or diseases. Although the three agonists had disparate targets, we intended to explore some shared targets for intervention in the progression of T2DM and MI, and 12 common targets were identified (Figure 3B), namely, AGTR1, AGTR2, CASP1, CCNA2, CCND1, CXCR4, EDNRA, F7, MME, REN, SCN5A, and SIRT1, suggesting that these 12 targets may contribute to the fundamental mechanisms of GLP-1RAs against T2DM and MI.

### Analysis of the PPI network of the related targets

**FIGURE 4:**
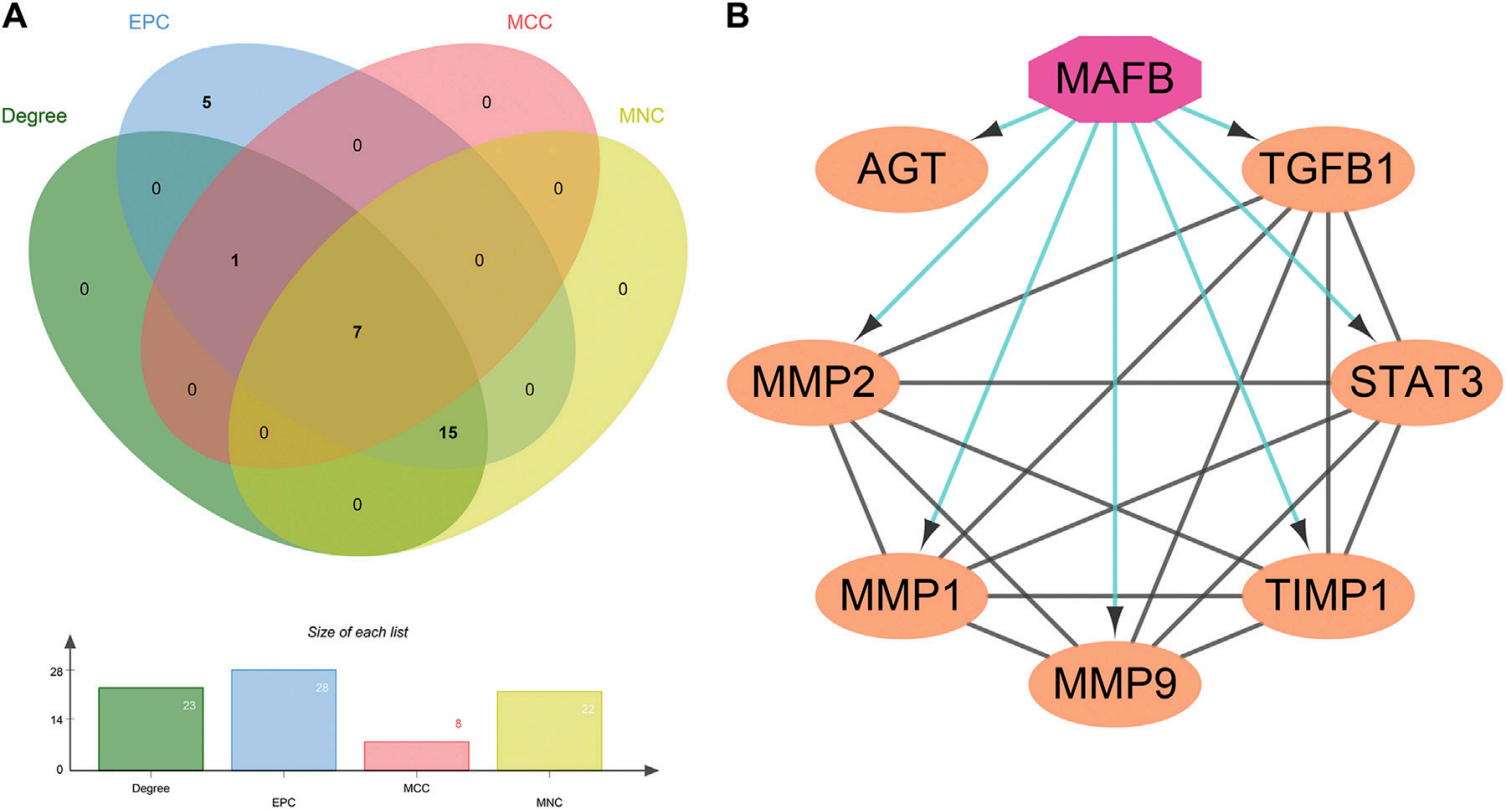
Seven core targets and one important transcription factor. **(A)** The core targets are obtained by the intersection of the four algorithms’ results: AGT, TGFB1, STAT3, TIMP1, MMP9, MMP1, and MMP2. **(B)** The interaction between the transcription factor (octagon) and seven core targets (ellipse) was analyzed and constructed by the Iregulon plugin in Cytoscape software.

The TSV file of the PPI network was loaded into Cytoscape software, and the MMC, MNC, EPC, and Degree algorithms in the “Cytohubba” plugin were used to calculate the core targets. Seven core targets were identified, including AGT, TGFB1, STAT3, TIMP1, MMP9, MMP1, and MMP2 (Figure 4A), suggesting that they may play a pivotal role in the PPI network of GLP-1RAs interfering with T2DM and MI.

Next, the Iregulon plugin was applied to find the direct transcription factors of seven core targets, and the normalized enrichment score (NES) was calculated and used for ranking purposes (Table 2). The higher the NES value, the better the confidence. The transcription factor MAFB had the highest transcription target number and NES value simultaneously (target number = 7; NES = 7.802) (Figure 4B). The Iregulon plugin detects transcription factors using more than one thousand ChIP-Seq tracks, providing highly credible results. Therefore, we suggest that MAFB may positively contribute to the beneficial effect of GLP-1RAs in reducing MI in T2DM patients.

**TABLE 2:** The top ten transcription factors (ranked by NES) related to six core targets.

Rank	Transcript factor	NES	Targets number	Motifs/Tracks
1	MAFB	7.802	7	11
2	ATF6	7.084	5	10
3	POU3F4	5.922	4	4
4	CBFB	5.803	3	3
5	CEBPA	5.719	4	10
6	FOXO1	5.684	3	8
7	FOXA1	5.561	6	14
8	NKFB1	5.536	4	6
9	SLC18A1	5.516	2	1
10	POU4F3	5.373	3	8

**FIGURE 5:**
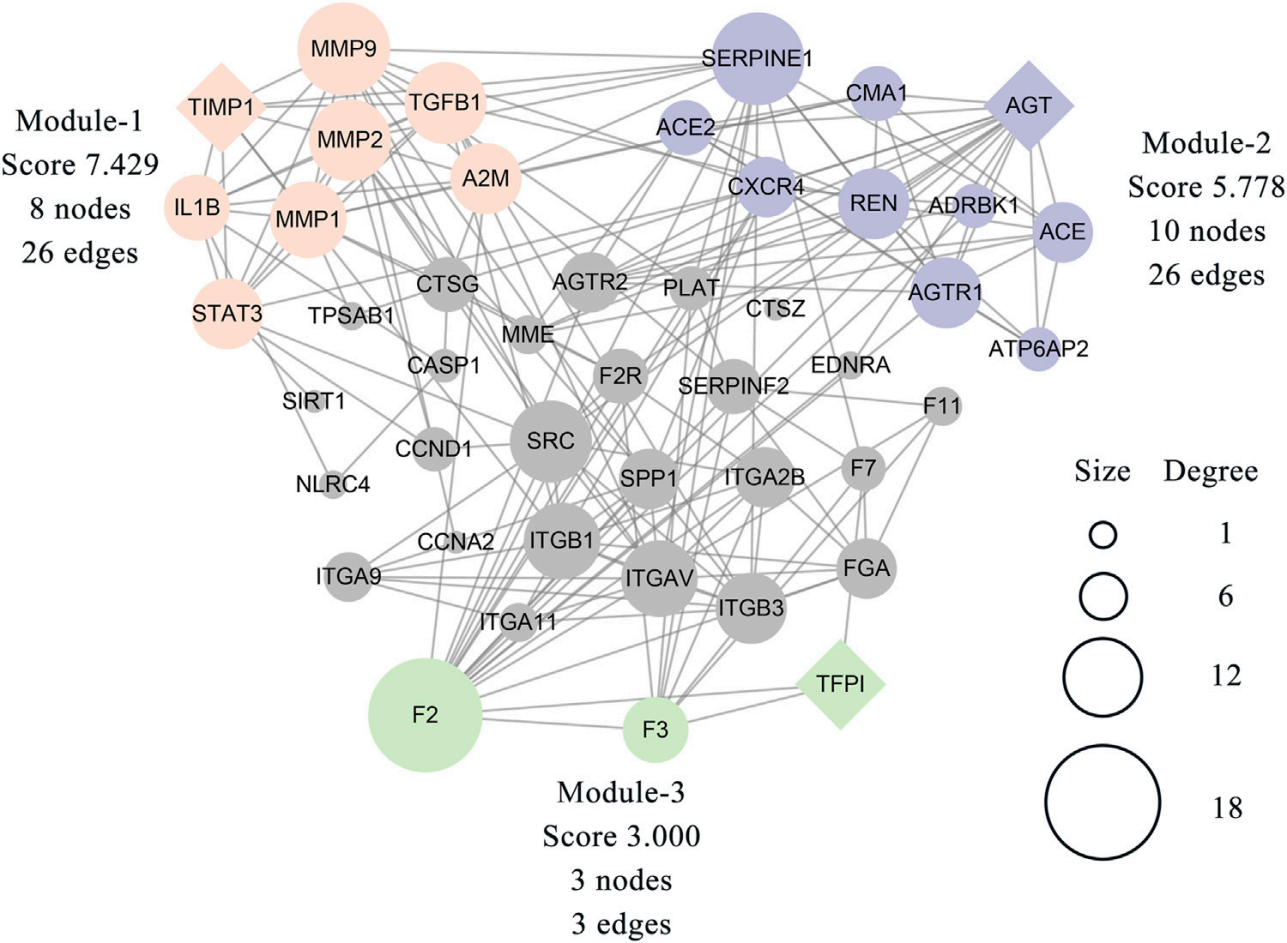
Three modules were clustered from 46 related targets of the PPI network and distinguished by different colors. The module-1 has top clustered strength according to the score. The size of each node is proportionate to the degree value of the node. The diamond-shaped nodes in each module represent the seed node with the highest weight.

The MCODE plugin was then used to predict the modules in the PPI network. The targets were clustered into three modules. Each module represents a densely connected region of the molecular interaction network (Bader and Hogue, 2003). Detailed characteristics of the modules are shown in Figure 5. Moreover, three seed nodes, TIMP1, AGT, and TFPI (marked by a rhombic shape), had the highest weights in the respective modules. KEGG analysis was performed using the Enrichr web tool. The three top-ranked terms in each module were the AGE-RAGE signaling pathway in diabetic complications (module 1), the renin-angiotensin system (RAS) (module 2), and complement and coagulation cascades (module 3). KEGG results are presented in Table 3.

**TABLE 3:** The top three terms (ranked by Odds Ratio) from KEGG pathway enrichment analysis for each module.

Module	KEGG term	Odds ratio	q value	Genes
1	AGE-RAGE signaling pathway in diabetic complications	207.25	3.57E-06	TGFB1; IL1B; MMP2; STAT3
1	Relaxin signaling pathway	158.94	4.99E-06	TGFB1; MMP1; MMP2; MMP9
1	Bladder cancer	315.06	1.28E-05	MMP1; MMP2; MMP9
2	Renin-angiotensin system	2912.88	4.16E-18	ACE2; ACE; CMA1; ATP6AP2; AGTR1; REN; AGT
2	Renin secretion	204.36	3.22E-07	ACE; CMA1; AGTR1; REN; AGT
2	Diabetic cardiomyopathy	99.96	3.22E-07	ACE; AGTR1; REN; AGT
3	Complement and coagulation cascades	59745.00	6.67E-07	F2; TFPI; F3
3	AGE-RAGE signaling pathway in diabetic complications	100.49	0.044	F3
3	Platelet activation	80.79	0.044	F2

### Global GO and KEGG enrichment analysis

**FIGURE 6:**
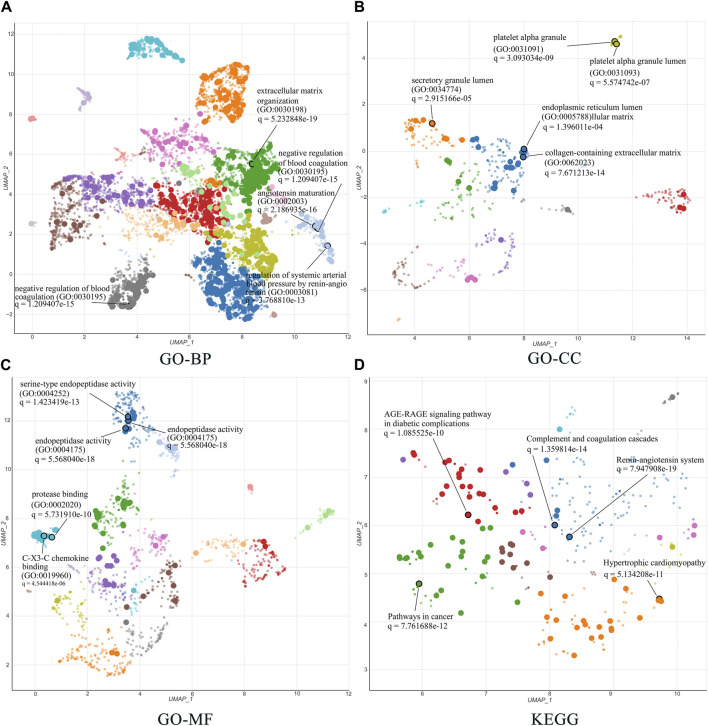
GO and KEGG enrichment analysis of 51 related targets *via* the Enrichr database. **(A–C)** Biological processes, cellular components, and molecular functions in GO biological annotation analysis. **(D)** KEGG pathway enrichment analysis entries. All results show the top five items according to the q value. The lower the q value, the higher the credibility.

The 51 related targets were uploaded to Enrichr for KEGG and GO enrichment analyses, and the top five terms ranked by q-value were selected for display. For the GO enrichment analysis, we identified the top five terms from BP, CC, and MF. The results are shown in detail in Figures 6A–C. First, in BP analysis, the top three terms were extracellular matrix (ECM) organization, angiotensin maturation, and regulation of angiotensin levels in the blood. The top three terms for CC were collagen-containing ECM, platelet-alpha granules, and platelet-alpha granule lumen. In the MF analysis, the top three terms were endopeptidase activity, serine-type endopeptidase activity, and serine-type peptidase activity.

The top five terms for global KEGG enrichment analysis are shown in Figure 6D. The detailed terms were RAS, complement and coagulation cascades, pathways in cancer, hypertrophic cardiomyopathy, and the AGE-RAGE signaling pathway in diabetic complications.

## Discussion

Complications related to diabetes result in 1.5 million deaths per year, and cardiovascular events are the primary cause of death (Collaborators, 2018). Several trials have demonstrated that GLP-1RAs protect diabetic patients from the occurrence of MI, but the underlying mechanism remains unclear. The present study provides novel and systematic explanations for how GLP-1RAs decrease the occurrence of MI in T2DM patients. We applied network pharmacology to predict 51 related targets between GLP-1RAs and diseases and filtered out seven core targets: AGT, TGFB1, STAT3, TIMP1, MMP9, MMP1, and MMP2. MAFB is an essential transcription factor that regulates the expression of all seven core targets. The GO enrichment terms mainly involved angiotensin, ECM, and platelets. For KEGG enrichment analysis, the related targets were principally enriched in RAS, complement and coagulation cascades, and the AGE-RAGE signaling pathway in diabetic complications.

Researchers have proposed various hypotheses to elucidate the therapeutic mechanism of GLP-1RAs in cardiovascular diseases. Many studies have consistently reported that several GLP-1RAs exert inhibitory effects on plaque formation, development, and rupture (Tashiro et al., 2014; Sudo et al., 2017; Wu et al., 2019; Li et al., 2020). However, evidence is insufficient to comprehensively explain the signaling pathways involved in the prophylactic effects of GLP-1RAs on MI. Our results are aligned with previous studies and hypotheses while also unveiling novel perspectives. After applying the CytoHubba plugin using a multi-algorithm, we identified seven core targets. Four targets (MMP1, MMP2, MMP9, and TIMP1) are associated with metalloproteinases. MMP1, MMP2, and MMP9 participate in ECM proteolysis, whereas TIMP1 acts as a metalloproteinase inhibitor that inhibits the function of MMP 1, MMP2, and MMP9 (Moore et al., 2012). The dynamic balance between MMPs and TIMP1 maintains myocardial ECM stability. Several reports have demonstrated that diabetes disrupts the balance of MMPs/TIMPs in the serum and related tissues (Li et al., 2013; Bastos et al., 2017; Zhou et al., 2021), which may significantly enhance the activities of MMPs and pathological remodeling of the vessel wall (Wang and Khalil, 2018), subsequently resulting in obstruction and ischemia. Several reports have shown that GLP-1, exenatide, and semaglutide reduce MMP expression (such as MMP1, MMP2, MMP9, and MMP13), which maintains intact fibrous caps and protects atheromatous plaque from rupture (Burgmaier et al., 2013; Garczorz et al., 2018; Rakipovski et al., 2018). GLP-1RAs may contribute to the reduction of atherosclerotic plaque instability and cardiac ECM degradation by maintaining the balance between MMPs and TIMPs.

The other three core targets identified were AGT, STAT3, and TGFB1. AGT-encoding angiotensinogen is an essential component of the RAS and participates in the regulation of blood pressure, body fluids, and electrolyte balance. Angiotensinogen undergoes two cleavages to form angiotensin II (Ang II), which has well-known adverse effects on the myocardium. A recent study showed that the mRNA expression of GLP-1R was considerably associated with the components of the renin-angiotensin-aldosterone system (RAAS) detected in epicardial and pericardial fat in patients with severe coronary artery disease (Haberka et al., 2021). However, the interactive regulation between GLP-1R and the RAAS is still unclear. TGFB1 mediates Ang II-induced myocardial fibrosis (Frangogiannis, 2019). Limited data have shown an inconsistent relationship between GLP-1RAs and TGFB1. Long-acting semaglutide decreased hepatic TGFB1 expression (McLean et al., 2021), whereas exendin-4 and liraglutide did not reduce TGFB1 levels in adipose tissue (Pastel et al., 2016; Pastel et al., 2017). Although GLP-1 has a beneficial effect on myocardial ECM remodeling (Robinson et al., 2015), the relationship between GLP-1RAs and TGFB1 in the heart is unclear and requires further investigation.

STAT3 responds to cytokines and growth factors. *Shiraishi et al.* demonstrated that GLP-1 induces macrophage transformation into the M2 phenotype, contributing to the beneficial effects of GLP-1 against diabetes (Shiraishi et al., 2012). A later study also provided consistent evidence that in the process of myocardial repair, STAT3 activation was a prerequisite for macrophage transformation to the reparative M2 phenotype (Shirakawa et al., 2018). Thus, the changes induced by GLP-1RAs contribute to a reduction in the size and instability of atherosclerotic plaques (Vinué et al., 2017), which could, to some extent, explain the lower MI mortality and incidence in patients using GLP-1RA.

The Iregulon plugin used in this study showed that the transcription factor MAFB may regulate all seven core targets, suggesting that it exerts crucial effects on GLP-RAs by interfering with T2DM and MI. In patients with T2DM, MAFB expression is significantly reduced (Guo et al., 2013). In contrast, overexpressed MAFB can upregulate some cell cycle regulators and subsequently promote human β cell proliferation (Lu et al., 2012). Many previous studies have considered MAFA to be a characteristic of human β-cell function, whereas this view is increasingly being challenged (Velazco-Cruz et al., 2019). Recently, it was suggested that MAFB could be regarded as an essential regulator of the human β-cell signature (Russell et al., 2020). However, only a few studies have tried to reveal the potential relationship between GLP-1 and MAFB. A recent study showed that exendin (9-39) accelerated the transdifferentiation from α cells to β cells by reducing MAFB expression in α cells (Zhang et al., 2019). Therefore, the relationship between GLP-1 and MAFB in β-cells warrants further investigation.

The MCODE plugin provides a practical clustering algorithm to identify the potential functional modules behind these targets. Three modules were obtained in this study and were subsequently subjected to KEGG analysis. The enrichment results mainly focused on the AGE-RAGE signaling pathway, RAS, and complement and coagulation cascades. These biological processes have been demonstrated to have a significant pathogenic relationship with T2DM and MI (Beckman et al., 2002; Husain et al., 2015; Yamagishi, 2019). Some researchers have provided evidence regarding the association between GLP-1RAs and the abovementioned results. GLP-1RAs, such as liraglutide and exenatide, attenuate RAGE expression in several cell types, especially under diabetic conditions (Zhang et al., 2016; Zhang et al., 2017; Zhang et al., 2020), suggesting that the downregulation of RAGE represents a potential mechanism of GLP-1RAs against T2DM. In module two, RAS was a significant KEGG-enriched item. GLP-1RAs play a competitive role in regulating RAS by inhibiting renin synthesis and increasing the inactive form of renin in blood circulation (Puglisi et al., 2021). The final module included three coagulation-related targets. To date, there is rare information on the direct influence of GLP-1RAs on the processes of coagulation cascades. Furthermore, hyperglycemia facilitates coagulation activation and invalidation of fibrinolytic activity in diabetic patients (Sechterberger et al., 2015). Thus, we hypothesize that the effect of GLP-1RAs on blood coagulation is mediated primarily through controlling blood glucose levels.

Several GLP-1RAs trials have demonstrated that these agonists reduce cardiovascular risk factors, including glycated hemoglobin (HbAc1) values, systolic blood pressure, and body weight (Marso et al., 2016a; Marso et al., 2016b; Hernandez et al., 2018). The median duration of these trials ranged from 1.6 to 3.8 years. Cumulative beneficial changes induced by GLP-1RAs contributed to a decrease in MI prevalence. However, the exact mechanisms underlying the contribution of GLP-1RAs remain unclear, as no cardiac tissue was detected in the trials. The GO and KEGG analyses showed that the enrichment terms mainly focused on ECM, coagulation, RAS, and endopeptidase. As most matrix metalloproteinases are elastase-type endopeptidases, mainly MMP2 and MMP9 (Shapiro, 1998), the dynamic balance of MMPs/TIMPs maintains the stabilization of the ECM; however, diabetes disturbs this balance and causes atherosclerotic plaque disruption, myocardial fibrosis, and remodeling. Four of the seven core targets were involved in this balance, and AGT and TGFB1 directly influenced fibrosis and remodeling. These pathological processes considerably induce cardiac death in diabetic cardiomyopathy and acute MI. Therefore, our results suggest that GLP-1RAs play a crucial role in regulating plaque stability, myocardial fibrosis, and remodeling. Other significant enrichment sets included coagulation, complement, and platelets. The burden of thrombus formation induced by coagulation and the complement system in the coronary artery mainly determines the MI area and clinical outcomes (Sianos et al., 2007; Sianos et al., 2010). Thus, inhibition of the coagulation cascades is an effective measure when plaque ruptures occur. To a certain extent, the related targets and enrichment terms involved in coagulation and complement provide a plausible mechanistic explanation for the fact that some GLP-1RAs reduce non-fatal MI in patients with T2DM.

## Conclusion

Our study provides a comprehensive investigation and analysis of the multi-dimensional effects of GLP-1RAs on preventing MI in patients with T2DM, which may be mainly mediated by interfering with specific targets, biological processes, and cellular signaling pathways related to atheromatous plaque, myocardial remodeling, and thrombosis. However, this study has some limitations as it lacks a series of experiments to prove the proposed hypothesis. Accordingly, further experiments and multi-omics studies are warranted to understand the comprehensive mechanism of GLP-1RAs.

## Data Availability

The original contributions presented in the study are included in the article/supplementary material, further inquiries can be directed to the corresponding author.

## Glossary

AGE: advanced glycosylation end products
AGT: angiotensinogen
AGTR1/2: angiotensin II receptor type 1/2
AMI: acute myocardial infarction
Ang II: angiotensin II
BP: biological process
CASP1: caspase 1
CC: cellular component
CCNA2: cyclin A2
CCND1: cyclin D1
ChIP-seq: chromatin immunoprecipitation-squence
CTSZ: cathepsin Z
CXCR4: C-X-C motif chemokine receptor 4
ECM: extracellular matrix
EDNRA: endothelin receptor type A
EPC: edge percolated component
F7: coagulation factor VII
GLP-1: glucagon-like peptide-1
GLP-1RAs: GLP-1 receptor agonists
GO: gene ontology
ITGA9/11: integrin subunit alpha 9/11
KEGG: Kyoto encyclopedia of genes and genes
MAFA: MAF BZIP transcription factor A
MAFB: MAF BZIP transcription factor B
MCC: maximal clique centrality
MCODE: molecular complex detection
MF: molecular function
MI: myocardial infarction
MME: membrane metalloendopeptidase
MMP1/2/9: matrix metallopeptidase 1/2/9
MNC: maximum neighborhood component
NES: normalized enrichment score
NF-κB: nuclear factor kappa B subunit 1
NLRC4: NLR family CARD domain containing 4
OMIM: online mendelian inheritance in man
ox-LDL: oxidized low density lipoprotein
PharmGKB: pharmacogenetics and pharmacogenomics knowledge base
PPI: protein-protein interaction
RAAS: renin-angiotensin-aldosterone system
RAGE: receptor for advanced glycosylation end products
RAS: renin-angiotensin system
REN: renin
SCN5A: sodium voltage-gated channel alpha subunit 5
SIRT1: sirtuin 1
SMILES: simplified molecular input line entry system
STAT3: signal transducer and activator of transcription 3
T2DM: type 2 diabetes mellitus
TFPI: tissue factor pathway inhibitor
TGFB1: transforming growth factor beta 1
TIMP1/2/3: tissue inhibitor of metalloproteinases 1/2/3
TSV: tab separated values
TTD: therapeutic target database.
